# Using theories of change to design monitoring and evaluation of community engagement in research: experiences from a research institute in Malawi

**DOI:** 10.12688/wellcomeopenres.13790.1

**Published:** 2018-02-08

**Authors:** Kate Gooding, Regina Makwinja, Deborah Nyirenda, Robin Vincent, Rodrick Sambakunsi

**Affiliations:** 1 Malawi Liverpool Wellcome Trust Clinical Research Programme, College of Medicine, Blantyre, 30096, Malawi; 2 Liverpool School of Tropical Medicine, Liverpool, Merseyside, L3 5QA, UK; 3 Independent learning and evaluation consultant, Sheffield, South Yorkshire, S8 9FH, UK

**Keywords:** theory of change, evaluation, community engagement, public engagement

## Abstract

**Background:** Evaluation of community and public engagement in research is important to deepen understanding of how engagement works and to enhance its effectiveness. Theories of change have been recommended for evaluating community engagement, for their ability to make explicit intended outcomes and understandings of how engagement activities contribute to these outcomes. However, there are few documented examples of using theories of change for evaluation of engagement. This article reports experience of using theories of change to develop a framework for evaluating community engagement in research at a clinical research organisation in Malawi. We describe the steps used to develop theories of change, and the way theories of change were used to design data collection plans. Based on our experience, we reflect on the advantages and challenges of the theory of change approach.

**Methods:** The theories of change and evaluation framework were developed through a series of workshops and meetings between engagement practitioners, monitoring and evaluation staff, and researchers. We first identified goals for engagement, then used ‘so that’ chains to clarify pathways and intermediate outcomes between engagement activities and goals. Further meetings were held to refine initial theories of change, identify priority information needs, and define feasible evaluation methods.

**Results:** The theory of change approach had several benefits. In particular, it helped to construct an evaluation framework focused on relevant outcomes and not just activities. The process of reflecting on intended goals and pathways also helped staff to review the design of engagement activities. Challenges included practical considerations around time to consider evaluation plans among practitioners (a challenge for evaluation more generally regardless of method), and more fundamental difficulties related to identifying feasible and agreed outcomes.

**Conclusions:** These experiences from Malawi provide lessons for other research organisations considering use of theories of change to support evaluation of community engagement.

## Introduction

Community engagement in research is widely recommended as an essential part of ethical and effective research practice ^1– 3^. In low-income settings in particular, community engagement serves a range of functions, including reducing exploitation and harm, promoting relevance, showing respect and stimulating public interest in science, as well as more instrumental functions such as supporting recruitment ^1, 4^.

However, community engagement is complex and challenging, and there is currently limited evidence on effective approaches ^5^. Reflecting this gap, there have been calls for further evaluation of community engagement to build the evidence base and help practitioners assess and revise their programmes ^4– 7^.

One approach recommended for evaluating community engagement involves developing theories of change (ToCs) ^4, 6^. The theory of change (ToC) approach originated in community development programmes in the United States in the 1990s ^8, 9^. ToC is part of a wider family of theory-driven evaluation approaches, which use an explicit model about how an intervention leads to its outcomes to guide evaluation design ^10, 11^. Theory-driven evaluations tend to focus on generative causation, examining the processes leading to intervention outcomes rather than relying on experimental logic, and they emphasise the role of context in contributing to varied intervention outcomes ^10, 12^. Other theory-driven designs include approaches such as process tracing and realist evaluation, which can be used in combination with ToCs ^10, 12, 13^.

A ToC sets out intended outcomes of an intervention and the steps between intervention activities and these outcomes, indicating underlying assumptions about how activities are expected to work ^6, 14, 15^. ToCs are often depicted visually, through a diagram showing pathways from activities to impact ^14, 15^. Beyond these core ideas, however, there are diverse interpretations of the ToC approach, with different purposes, designs and levels of complexity ^6, 14, 15^. For example, while some ToCs depict a chain from activities to outcomes, more advanced approaches explore the mechanisms and contexts that generate different outcomes, drawing on the logic of realist evaluation ^16^.

Since the 1990s, ToCs have become increasingly popular within international health, development and research, for both design and evaluation of interventions ^14, 15, 17^. ToCs have several potential benefits for evaluation. In particular, they can provide an organising framework that indicates areas for evaluation at the level of ultimate outcomes and intermediate processes, and they can be more flexible than a logical framework approach, facilitating consideration of multiple causal pathways ^15, 18^. The current popularity of ToC approaches may also reflect their potential appeal for both those interested in linear planning and those concerned to address complexity: ToCs can be deployed much like a logical framework to make project planning logic explicit, or to draw attention to context and the constantly changing environment, requiring their use as an iterative planning and learning tool ^18, 19^.

A growing body of evidence examines use of ToCs for health interventions, international development and research uptake ^13, 14, 17, 18^. However, there is little documented experience of using ToC for evaluating community engagement in research (we found only one detailed report ^20^). Given the attention to evaluating community engagement and the potential advantages of a ToC approach, examples of using ToCs have value for informing their future use. This article reports experience of using ToCs to plan evaluation of community engagement at the Malawi-Liverpool-Wellcome Trust Clinical Research Programme (MLW). Our aim is to provide lessons for other organisations interested in the ToC approach by sharing an honest and practical account of the process, advantages, and challenges.

## Methods

### Setting

MLW is a clinical research programme based in Blantyre, Southern Malawi.

Community and public engagement is a core area of MLW’s work. The Science Communication Department was established in 2008 and now runs a range of engagement activities. Key initiatives include a weekly radio programme about health and research, a journalist in residence initiative, community advisory groups, community film shows and discussions about research, internships, career talks, and a science exhibition project that involves a permanent exhibition at the MLW premises and an outreach exhibition taken to rural schools and communities. The Department also supports community engagement activities linked to specific research studies, particularly community meetings to discuss research plans. There are seven people in the Department: five practitioners responsible for developing and implementing engagement activities, a Monitoring and Evaluation Coordinator, and the Department manager.

Evaluation initially focused on the radio and exhibition projects. To support improvement across the Science Communication programme, the Department wanted a clear monitoring and evaluation (M&E) plan that covered the full range of engagement activities. The ToC approach was selected as a way to develop the M&E plan. In particular, we felt ToCs would support evaluation focused on intended outcomes, rather than just activities. The existing Science Communication strategy indicated ‘supporting ethical research practice’ as the overall mission, but designing outcome-focused evaluation required more detail on the pathways through which engagement activities were expected to contribute to this mission. Using ToCs held promise as a way to clarify intended outcomes and steps towards progress.

### The process of developing theories of change and evaluation plans

A four-step approach was used to develop evaluation plans based on ToCs: clarifying the purpose of evaluation, clarifying the aims of engagement activities, identifying information needs, and identifying methods to meet those information needs. We describe this process using the exhibition project as an example.

### Step 1: Clarifying the purpose of M&E

M&E can serve different functions, including driving programme learning, accountability to donors, demonstrating success or influencing policy ^6^. The aims of M&E shape the appropriate approach, for example by indicating relevant stakeholders and the balance between timeliness and comprehensive data. Consequently, our first step was confirming the purpose of M&E through discussions within the Science Communication Department.

For the Department, the primary purpose of M&E is to strengthen effectiveness of MLW’s public and community engagement by supporting learning within the team about gaps and areas for improvement. To support this focus on informing immediate team practice, we drew on ideas from utilization-focused evaluation, in particular engaging primary intended users throughout the process of designing M&E frameworks to promote relevance and ownership ^21^. In line with this, input from Science Communication practitioners - the team members responsible for developing and implementing engagement activities - has been central to M&E design.

### Step 2: Developing theories of change

The ToCs that formed the basis of the M&E strategy were developed through a one-day workshop for members of the Science Communication Department. This was facilitated by two social science researchers and the Science Communication Manager. In planning the workshop, we drew on ToC guidelines to identify potential exercises ^22– 26^, and adapted suggested approaches to suit our needs and resources. When ToCs are used to design interventions, they tend to start by defining intended goals and working backwards to identify appropriate activities. As MLW engagement activities were already underway and our focus was evaluation, we instead used approaches that worked forwards from activities to intermediate outcomes and overall goals. We consider limitations of this approach in the discussion.

The workshop started by explaining the idea of ToCs. To maximise accessibility for practitioners unfamiliar with the concept, we downplayed the potential complexity of ToCs, and emphasised a purpose of clarifying how engagement activities lead to the changes we want to see. The alternative terminology of ‘impact pathways’ helped communicate the central ideas more easily (see guide from BetterEvaluation).

**Figure 1.**
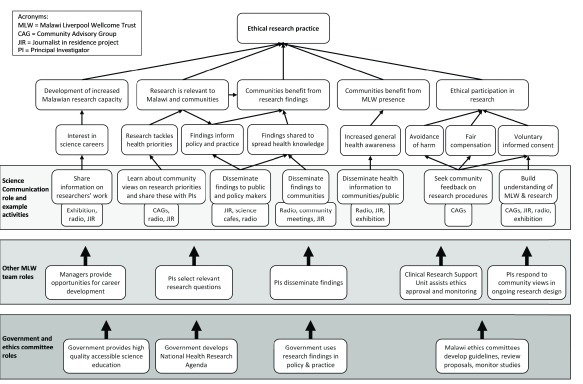
Overall theory of change for Science Communication activities.

The first group exercise examined the overall purpose of engagement activities, to clarify the stated mission of ethical research practice. Participants wrote ideas about the ultimate aims of engagement activities on post-it notes, which we then grouped and discussed. This proved a challenging exercise: practitioners immersed in daily activities struggled to think about higher aims, and answers tended to focus on intermediate outcomes such as two-way engagement. A productive discussion resulted in a set of higher level goals linked to ethical practice, including contributing to research capacity in Malawi, promoting relevant research, promoting societal benefit from research findings, and supporting ethical research participation (including aspects such as informed consent). This broad interpretation of ethical practice, extending beyond the ethics of individual participation, is in line with guidelines on research ethics in low-income countries ^27– 29^. We used a whiteboard to depict these ethical goals in a diagram, providing an overall ToC for Science Communication ( Figure 1). Recognising that these goals depend on actions beyond the Science Communication Department, we also indicated responsibilities of other actors. For example, community engagement can contribute to informed consent by supporting understanding of research, but informed consent also depends on action by researchers and ethics committees. This ToC is a partial and preliminary representation of Science Communication outcomes and roles, rather than our definitive understanding.

The second exercise developed ToCs for each major engagement activity by using ‘so that’ chains ^25^. These chains identify steps through which particular activities lead to the overall goal by starting from the activity, asking why it is being done, and then asking why that intermediate outcome is important; for example, we invite children to the exhibition *so that* children learn about science *so that* they become more interested in science *so that* they choose science subjects at school *so that* they study science at university *so that* they can become scientists *so that* Malawi’s scientific capacity is increased. Chains can involve several pathways and multiple outcomes. For example, exhibition attendance might increase understanding of research both directly and through students sharing their learning with others, and increased understanding of research could support both informed consent and interest in science careers. To develop ‘so that’ chains, we worked in groups of two or three (including the practitioners responsible for the specific engagement activity), and used post-it notes to depict the chains on flipcharts.

As part of this exercise, we planned to identify assumptions about conditions needed to enable outcomes, such as high quality, accessible science education. The involvement of a social scientist conducting research on engagement provided a critical perspective that assisted in identifying assumptions. However, although some ideas were noted, completing this component proved difficult within time constraints.

### Refining theories of change

Finalising ToCs within a one-day meeting was unrealistic. To refine and clarify draft ToCs, we held follow-up meetings with staff leading on each activity, facilitated by a social scientist and the M&E Coordinator. Meetings involved in-depth discussion to reflect carefully on aims and factors affecting these aims. Asking practitioners what they hoped to see and whether intended outcomes are always achieved helped to support reflection and identify clearer pathways and assumptions.

**Figure 2.**
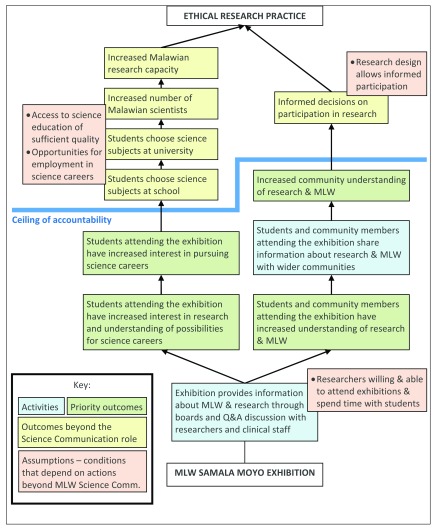
Theory of change for the permanent science exhibition, ‘Samala Moyo’.

Figure 2 provides an example of the science exhibition ToC. This shows the exhibition activity at the base, with two main impact pathways: one related to building understanding of MLW and research, which in turn may support informed engagement with research, and one related to building interest in research that may eventually support a science career. The diagram also indicates conditions beyond the remit and capacity of Science Communication but required to achieve these goals, including access to high quality science education and research employment opportunities. These conditions mean the Science Communication Department can only contribute to higher level outcomes and is not responsible for achieving them, an idea shown by the ceiling of accountability line ^15^. Identifying this ceiling of accountability follows a wider recognition within evaluation thinking about the value of focusing on contribution rather than attribution ^30^.

More time could have been spent perfecting ToC diagrams and adding detail on assumptions and contextual factors. However, ToC guidance emphasises that ToCs provide a basis for planning and discussion and should be adapted in response to new information ^18^. We followed this approach, and continued changing the diagrams as later M&E planning prompted further reflection.

### Step 3: Identify priority information needs

The next step was identifying information to collect through M&E. As resources are always limited, M&E should focus on priority information likely to be most important for strengthening effectiveness ^21^.

In line with our interest in supporting team learning and the utilization-focused evaluation approach, information needs were prioritised through discussion with the staff leading each engagement activity. Discussing how data would be used for programme decision-making helped to focus on the most actionable information. For example, exhibition practitioners felt information on school attendance could be used to follow up schools that do not attend, to understand and address any barriers to participation.

ToC diagrams provided a basis for identifying information needs: reviewing the pathways helped indicate which steps were critical to success or had particular uncertainties. For example, with the exhibition, gaining an understanding of research and interest in science careers are likely to be important steps in moving to a science career. In deciding key steps, we also drew on the team’s experience and previous research on MLW’s engagement ^31, 32^. We also included aspects of the implementation process not explicit in ToC diagrams but identified as important for ongoing programme decisions or as key factors affecting outcomes. For example, previous evaluations suggested that students gain more from the exhibition when there are adequate facilitators and a researcher attends, so practitioners wanted to track these components. Identification of information needs also drew on standard evaluation components such as reach, reasons behind outcomes and unintended effects ^6^ (see BetterEvaluation page on identifying unintended results).

We focused on lower level outcomes where Science Communication has more control, rather than ultimate outcomes above our ceiling of accountability. For the exhibition, this means more emphasis on assessing interest in science than on informed consent and research capacity. However, we included some higher level outcomes (particularly moves to university), to explore barriers or enablers that might be addressed through future activities.

Our original exhibition ToC did not indicate impacts on researchers. Engagement at MLW aims at two-way dialogue between communities and researchers, sharing information on MLW work but also seeking community feedback and ideas to inform MLW research. This community feedback is a particular priority for activities such as the Community Advisory Group. However, researchers involved in the exhibition may also receive feedback on their work or be influenced in other ways. Consequently, we included an exploratory question about effects of the exhibition on researchers within the information needs. Table 1 indicates the information needs identified for MLW’s permanent exhibition.

**Table 1.** Information needs and data collection methods for evaluating the Samala Moyo science exhibition.

Information needs and areas for analysis	Methods							
	Attendance monitoring spreadsheet	Participatory assessment at the exhibition	Focus groups with students	Questionnaire for students	School records	Interviews with school leavers	Interviews with teachers	Focus group with researchers
1. Attendance: - Which schools attend and why? - Are there barriers to attendance? - Do we reach students in poorer neighbourhoods? - Is there a balance of boys and girls?	X							
2. How is the exhibition implemented: - Do researchers speak to students? - How much time do students spend at the exhibition? - How many facilitators are there? - Any difficulties with implementation?	X							
3. Exhibition format and approach: - Do students enjoy the exhibition? - Are they satisfied with the format & content? - Do they understand the content?		X	X					
4. Do students attending the exhibition gain more understanding of research and MLW? - Why or why not? - How does this vary between students and schools?		X	X	X				
5. Do students attending the exhibition gain more interest in science careers? - Why or why not? - How does this vary between students and schools?		X	X	X				
6. Do students attending the exhibition choose science subjects at school? - Why or why not? - How does this vary between students and schools?			X	X	X			
7. Do students attending the exhibition move to university science courses? - Why or why not? - How does this vary between students and schools?					X	X	X	
8. Are there unexpected effects, positive or negative?			X			X	X	
9. How does the exhibition affect researchers?								X

### Step 4: Identify M&E methods to address information needs

Having agreed information needs, we then identified methods to collect this information. In selecting methods, we balanced a pragmatic understanding of available time and resources against a concern for adequate rigour to support confidence in the conclusions, including through triangulation and examining views of different stakeholders. Methods were agreed through discussion between the M&E Coordinator, a social scientist and engagement practitioners. Table 1 summarises methods against information needs for the exhibition. A more detailed table used by the team also shows the sample, timing and person responsible for each method. We planned a combination of methods, including spreadsheets to record attendance and implementation processes, participatory exercises with students, qualitative interviews and focus groups, a short questionnaire and comparison against school records of subject choice and graduation. For methods such as the attendance spreadsheet, the sample is all schools invited to the exhibition; for more intensive methods such as the questionnaire and focus groups, the sample will be four to eight schools, covering primary and secondary and varied socioeconomic settings.

Data collection tools are now being piloted. To support use of data by practitioners, we aim to maximise collection and initial analysis of data by engagement practitioners. External evaluators can bring new perspectives and credibility with some stakeholders, but our priority of team learning makes it useful for practitioners to be fully involved in ongoing data generation and analysis (see BetterEvaluation page on deciding who will conduct evaluations). Increasing the practitioner role in data collection and analysis also avoids overburdening the M&E Coordinator.

We plan to revise methods through experience of their feasibility and value and in response to emerging information needs.

### Discussion with peers to refine plans and challenge ideas

Throughout the four steps above, plans were discussed with internal and external colleagues to benefit from their suggestions. We shared draft ToCs and M&E plans with experts in M&E and engagement in other countries, through email, Skype and presentations at two workshops on community engagement (the Global Health Bioethics Network 2016 Summer School, and the GHBN/MESH 2017 Evaluating Community Engagement workshop). These discussions challenged and refined our thinking about intended goals and impact pathways. In particular, they pointed to the importance of considering impacts on researchers, the difficulty of attaining some higher level outcomes, and the value of less instrumental outcomes such as enjoyment among participants, aspects discussed further below.

## Results

### Benefits of the ToC process

Using ToCs and the steps outlined above had several benefits for the team and M&E design. The main value of ToCs was facilitating identification of M&E information needs, our primary rationale and a core purpose emphasised in ToC guidance ^22^. Developing ToCs provided a systematic way to identify relevant evaluation questions, by indicating outcomes and stages in the impact pathway where information was needed.

However, the process of developing ToCs had additional benefits. In particular, ToC development was motivating for engagement practitioners and provided an opportunity to reflect and recall the bigger purpose of daily work. Involvement of practitioners in each step of M&E planning and the focus on meeting practitioner information needs also increased interest in M&E among team members. There is now high demand for M&E findings and attention to M&E when new activities are planned, and more enthusiasm for sharing M&E responsibilities.

The reflection during ToC development also provided an opportunity to review activities and identify changes needed to support intended outcomes. For example, when discussing ToC for the journalist in residence and radio programmes, we realised these activities could provide valuable feedback for researchers, but that systems for consistently capturing and sharing this feedback to all MLW teams were not adequate. Consequently, feedback systems are now being strengthened.

This value of ToC for critical reflection and learning is sometimes constrained by requirements to demonstrate results and follow fixed plans agreed with donors ^33^. The MLW context of a core-funded institution and funder that allows considered revision of activities was important in enabling M&E focused on learning and use of ToC to adapt activities.

### Challenges and dilemmas

In designing ToC and evaluation strategies, we encountered several difficulties and uncertainties. These involve practicalities and more conceptual difficulties related to outcomes and strategies.

In relation to practicalities, a key issue is time for busy practitioners to be fully involved in ToC development and M&E planning (a common challenge for M&E, whatever the approach). A one-day workshop was too short to develop ToCs, particularly when covering several engagement activities. Further meetings helped to refine ToCs, but even two or three follow-up meetings were not enough to think through all assumptions and existing evidence. Time may have been a particular challenge because staff were unfamiliar with ToCs; the process may be faster once staff feel confident with the approach. Staff turnover also increased the time needed, as additional meetings were required to familiarise new staff with ToCs and seek their input to M&E plans.

The details of designing ToC diagrams also raised dilemmas and practical difficulties. One issue was whether intermediate outcomes should be framed as actions (such as students share information on research with wider communities), or outcomes (such as communities understand MLW research). Certain steps (such as students sharing information) might also be considered as either actions or assumptions. We found different ideas on this in ToC guidelines. In practice, this did not affect identification of information needs; whether something is labelled as an action, outcome or assumption does not determine whether it needs evaluating. Actual design of ToC diagrams also proved a challenge. Microsoft Word 2016 software proved cumbersome for neat diagrams, and engagement practitioners were unfamiliar with some features. A range of free software tools are available for designing ToC diagrams, and we would explore these options in future (see BetterEvaluation page on ToC software and article on Software for ToC).

A further practical challenge relates to resourcing and implementing M&E plans. Despite attempts at prioritisation, the ToC exercise produced an ambitious set of information needs and methods. These original plans were scaled down for feasibility. A selective approach is also needed because the Science Communication Department regularly develops new activities. Each new activity comes with an expectation of M&E; indeed, a side effect of the welcome enthusiasm for M&E is demand that cannot easily be met. Full M&E across all engagement activities would be unfeasible and inefficient, so we aim to make M&E proportionate ^6^, focusing on activities that involve greater investment or uncertainty about effects, or that are particularly crucial for ethical practice. Practitioner involvement in data collection and analysis is also designed to enhance feasibility, as relying on a single M&E coordinator for all evaluation tasks is unmanageable.

Conceptual challenges related to the identified outcomes and impact pathways. In relation to outcomes, one issue is that developing a ToC and attempting to link activities to high level goals encouraged a focus on longer-term outcomes that may be unrealistic given the constraints of the setting and scale of activities. For example, promoting science careers is an important goal for MLW, but the contribution of one morning at an exhibition to a child’s career choices and future achievements is inevitably minimal. Focusing on higher level goals also detracts attention from more immediate, short-term but arguably important outcomes such as enjoyment among students. The ToCs also gave less explicit attention to intrinsic purposes of engagement, such as showing respect. Such purposes are an inherent part of engagement ^1^, but their intrinsic nature makes them less obviously placed within causal impact pathways. Similarly, important but less tangible outcomes such as enhancing the quality of relationships ^6^ were lost in the focus on more visible steps. Recognising these difficulties, we included more immediate impacts (such as enjoyment) within M&E plans, as well as unintended consequences, and may in time revise the ToCs and refocus data collection away from long-term, instrumental outcomes such as career impacts. A focus on tangible, instrumental outcomes is not an inherent feature of ToCs, and evaluation frameworks and methods could be developed to examine more subtle and intrinsic goals ^6^.

Another challenge related to outcomes involves the diverse and potentially conflicting aims for engagement held by different stakeholders. The outcomes and pathways used to plan M&E were developed by the Science Communication team. Community members, researchers and other stakeholders may have different ideas about intended outcomes. Ideally, ToCs should be developed through input from these different stakeholders ^13, 15^. We followed an internal process to allow initial experimentation with the ToC approach, but hope to broaden discussion around goals and pathways beyond the team. Even restricted to internal discussion, ToC development helped to clarify potential tensions around goals, for example whether activities are designed to encourage informed decisions on participation or high recruitment.

Discussion about M&E methods and early evaluation findings also highlighted the need to be more specific about some intended outcomes. For example, our exhibition ToC indicated interest in science careers as one outcome, but is this biomedical science or also social science, and does it include science careers outside research such as engineering? Further, while MLW aims to develop research capacity, it may be a positive outcome for an individual student and indeed Malawi if a student who attends the exhibition becomes a doctor, civil servant, or focuses on caring responsibilities rather than employment. The outcome of increased understanding of research also requires clarification to specify which aspects of research we hope people will understand. Identifying higher goals helps to explore these details. For example, when understanding of research relates to informed consent, aspects such as the distinction between research and healthcare or the centrality of voluntary consent may be key; when understanding relates to supporting research careers, the focus may be on the potential contribution of research or the daily life of a researcher.

Identifying impact pathways also raised challenges, in particular finding intermediate steps that were both necessary and sufficient to support ultimate outcomes. This was particularly evident in relation to science careers. To assess progress, we need intermediate steps between promoting interest in research and increased numbers of research scientists. Potential intermediate outcomes include choosing science subjects at school and university (as indicated in Figure 2). However, there are multiple pathways to a career in science; for example, MLW staff come from backgrounds in numerous disciplines and from careers as clinicians or NGO staff. Similarly, students who choose science at school may move onto successful careers in other professions. Consequently, subject choice is not a reliable indicator of future career, complicating assessment of programme outcomes.

These dilemmas partly reflect the inherent complexity of engagement, with multiple goals and uncertain impacts ^1, 6^. Some uncertainties can be resolved through further discussion about intended outcomes with different stakeholders, and through use of M&E findings to enhance understanding of potential pathways and effects. While we have not yet answered all our dilemmas, ToCs were valuable for raising awareness of uncertainties, in turn supporting our ongoing process of clarifying aims and refining engagement strategies.

## Limitations

Our approach to using ToCs has been relatively simplistic. More complex ToC incorporate more attention to assumptions, unintended effects, contextual factors, barriers, ways to overcome barriers and indicators ^15, 16^ (see guide from BetterEvaluation). In addition, as above, we focused on discussion within the team, but ToC can be more effective when developed through dialogue with different stakeholders ^13, 15^. A further limitation relates to the stage at which we developed ToCs. Ideally ToCs should be used during strategy development to identify goals and then activities needed to meet these goals ^15, 26^. We introduced ToCs once engagement activities were underway, an overall team goal had been identified and a written strategy existed. This created a retrospective approach of fitting activities to the goal, with attached risks of suggesting unrealistic outcomes and missing alternative goals. In addition, while developing ToCs indicated some ways to improve engagement activities, using ToC once activities were underway encouraged relatively minor adaptation. Introducing ToC as part of M&E planning may also limit team awareness of the potential value of ToCs to plan activities and communicate strategy.

In part, this ‘ToC lite’ approach ^26^ reflects our aim of developing M&E plans rather than strategy planning, and our interest in trying out ToC as people new to the approach. It also reflects our resources: the process outlined here involved minimal funding (lunch for a one-day workshop); we did not hire professional facilitators or external venues. Strictly following processes set out in ToC guidelines would have exceeded financial and time constraints.

Our approach may also have been strengthened through additional expertise. Within our team, one social scientist had developed programme theories and read ToC guidelines, but ToCs were a new concept for others. Seeking feedback from external colleagues with ToC experience gave us confidence to proceed, but some dilemmas and challenges may have been avoided with more ToC experience.

While our process was small-scale and further steps could be taken, the ToCs at least partially meet three core ToC quality criteria of usefulness, clarity and ownership ^13^: the diagrams provided a clear and valuable framework for M&E planning, and involving practitioners contributed to their interest in both ToCs and M&E.

## Conclusion

This article contributes to the literature on evaluation of community engagement by reporting experience of using ToC to develop evaluation plans. Our experience suggests the potential value of even a minimalist, low-resource approach to ToCs. However, it also highlights challenges, including the complexity of identifying outcomes and pathways, and the risk of creating overambitious evaluation plans. Key practical lessons include the value of discussing draft ToCs and evaluation plans with external experts, to seek advice and challenge thinking, and the need to adjust approaches to fit practitioners’ availability. The volume of guidance and critical literature on ToCs can be overwhelming. Overall, we would encourage other engagement practitioners to avoid undue concern about rigidly following all aspects of this guidance, to select ideas that seem useful and feasible, and to enjoy experimenting with the ToC approach.

## Data availability

*All data underlying the results are available as part of the article and no additional source data are required.*
