# Supplementary material for: Using theories of change to design monitoring and evaluation of community engagement in research: experiences from a research institute in Malawi
Source: Wellcome Open Res. 2018 Feb 8;3:8. [Version 1] doi: 10.12688/wellcomeopenres.13790.1 (PMC5824328; doi:10.12688/wellcomeopenres.13790.1)
Supplement: Peer review history

# Peer review: Referee response for version 1

**DOI:** 10.21956/wellcomeopenres.14989.r30732

**Published:** 2018-03-01

**Authors:** Chris Barnett

**Affiliations:** 1 Centre for Development Impact (CDI), Institute of Development Studies, Brighton, UK

Overall, this article provides a useful and clear description of the process of applying ToCs to community engagement for research in Malawi, and highlights some of the challenges. The article also makes clear that it was a scaled-back version of ToCs that was applied - and is sufficiently humble and transparent about the constraints in applying such an approach in this context. The paper is helpful in providing insights about how ToCs can be practically applied. There are however a number of areas where it could be improved: 

- Defining ‘engagement’ in this context: The article assumes that the reader understands what is meant by community engagement and how it is being specifically applied here. In particular, while the article concludes (p10) that “ *this article contributes to the literature on the evaluation of community engagement*” there is limited reference to origins and definitions in this particular field. While accepting that there might be limited literature on *actual evaluation* of engagement for health research, there are adjacent literature on evaluating ‘community engagement’ that could be relevant and insightful.
- The four steps in applying ToCs in this case are clearly written, although these are more descriptive than analytical. This takes up a large proportion of the paper (pages 4-8), and could benefit from being more critically reflective. While admittedly the next section on “Results” (pages 8-9) pulls out some of the challenges, it tends to focus (though not exclusively) on practical time and resource challenges. For instance, I’m surprised not to learn more about: (1) The trade-offs and tensions encountered during the workshops: e.g. the ethical avoidance of harm vs. the more positive ethical promotion of societal benefits; tensions between informed consent (individual research participation, page 4) and the more public debate of data and findings (societal benefits); etc. (2) Political considerations, as in small ‘p’ political around competing views and interests. While admittedly the paper highlights that other perspectives weren’t able to be included (researchers, community members, etc.), I do wonder if there could be more reflection of different viewpoints and competing interests around some aspects of the ToC outcomes and assumptions? In short, the diagrams and ToC steps seem very neat and free of tension, yet usually ToC workshops lead to interesting discussions, different points of view, and new insights – can more of this be shared?
- The paper states that, “ *there are few documented examples of using theories of change for evaluation of engagement*”. This should be more accurately caveated with the fact that there are few *publicly available* examples *in this field* (which first needs defining, see first comment). ToCs are widely used across lots of fields, and they are mostly written up in grey literature (project documents, workshop notes, etc.). I agree that there is a lack of more critically reflective (and academic) papers that assess the application of theories of change, but would be less sure that they are not documented for internal purposes given that it has become fairly standard practice in international development.
- Table 1 on methods is very cursory, and it is not clear what this really adds to the paper. There is no in-depth discussion of how different methods are being used to capture and analyze different aspects of the ToC, ways of triangulation and verification between different data sources, etc. There may not be space to do so in this paper, but that does raise questions about why Table 1 is included? What does it really add?
- The ToCs outlined (in the diagrams) and the four-step process seem rather content-free to the actual research topic being undertaken. Maybe this is because this is a generic framework for the Science Communication Department – and so this observation is somewhat unfair. However, I do wonder if particular type of research with particular target groups (different communities, urban / rural, men / women, young / old, etc.) might give rise to additional insights.
- The “Results” section (pages 8-9) might more accurately be described as “Reflections” or “Discussion”.
- And lastly, it would be interesting to see a “Part II” of this paper, focusing on what happened when this was applied? Did researchers and community members have very different views? Was it as linear / predictable as it seemed at the workshop, or did practice thrown up new surprises? Was it as useful as originally thought?  Did the ToC change end up evolving and being substantially different? Did it work better for some types of research topic / communities than others? Etc.

I have read this submission. I believe that I have an appropriate level of expertise to confirm that it is of an acceptable scientific standard.

# Peer review: Referee response for version 1

**DOI:** 10.21956/wellcomeopenres.14989.r30663

**Published:** 2018-02-21

**Authors:** Alun Davies

**Affiliations:** 1 Kenya Medical Research Institute-Wellcome Trust Research Programme, Kilifi, Kenya; 2 Centre for Tropical Medicine and Global Health, Nuffield Department of Medicine, University of Oxford, Oxford, UK

***The article’s contribution to literature***

 It’s ironic that though the number of frameworks and guidelines for evaluating engagement have increased over the last two decades, the number of actual evaluations of engagement, or articles describing themselves as engagement evaluations, are remarkably rare. In this respect, this article by Gooding and colleagues is a rare and welcome description of how a standard framework for evaluating community initiatives/projects, the Theories of Change framework, has been applied to a set of community and public engagement activities. This article does not provide any evaluation data as such, and a justification of this is provided in the text, however, it provides a fairly clear and concise account of how the ToC framework and process were used in Malawi to reflect on and re-define the ethical goals of their engagement activities, and from these, to derive methods of evaluation. In this initial exploration of ToC by the group, the process was conducted by engagement practitioners and social scientists, however, the study points to an aspiration of extending this participation to include community practitioners. This is a commendable aspiration which would seem to align itself strongly with the participatory nature of engagement. This article provides a good contribution to the literature on community engagement.

***Comments for specific sections***

***Abstract***

 One of the novel innovations (unless it is a standard ToC methodological approach?) of this article, is the way in which ToC has been applied through 4 steps. It would be worth highlighting this and to outline these four steps in the method of the abstract.

***Introduction***

 The introduction is concise and provides a good overview of the context leading to the approach taken by the authors/practitioners. A short orientation (1-2 sentences) on why the evaluation of CE is complex and difficult, according to the literature, would be useful and would provide a justification of why a ToC was selected.

***Methods  ***

- The introduction provides a background on community engagement and moves onto the ‘methods’ section which describes MLW’s ‘Public’ (PE) and ‘community’ engagement (CE). It would be useful for the reader if the authors either describe the differences between CE and PE from the literature, or, provide a description of how they have defined the terms for their engagement and study. This could be in the introduction or methods section.
- Prior to the use of the ToC the authors describe the original engagement activities as having an overarching aim of ‘supporting ethical research’. I feel that this overarching aim does not provide enough of an understanding of why the activities, for example, a radio show were originally implemented? It may be useful to give a more in-depth description of some of the *original* specific goals of the individual activities (maybe a short table of the activities and their original specific goals) at the outset, and perhaps later in the discussion to give some discussion on how these goals changed after applying the ToC process. (If there were no original specific goals, then please ignore this comment.)
- A strength of the paper is the application of the ‘ceiling of accountability’, and the recognition/acknowledgement that CE can only make limited contributions to addressing many societal goals. A good example is the acknowledgement that science museum visits on their own, have a limited capacity to provide prolonged inspiration in science to school students, without the corresponding/complementary infrastructural support of a high-quality education.
- ‘Insider evaluations’ are vulnerable to a criticism of being susceptible to bias or lacking objectivity. The method, and description of the overall approach would benefit from more of an emphasis (a short 1-2 sentences) on the researchers’ positionality, or how the practitioners, in being the evaluators of their own activities addressed objectivity or limited bias. This could be through describing practitioner/researcher ‘reflexivity’ (this is discussed but not emphasised as being ‘reflexive’ in the conclusion), or through a sentence to describe the advantages of ‘insider’ evaluation – e.g. rich insights into the way activities were implemented, or a better understanding of the specific context, could be gained through insider knowledge as opposed to through an external evaluator.

***Results***

 This section could equally be described as a ‘Discussion’ section. Some additional areas which could be reflected upon in the discussion section:

- The practitioners prior understanding of the ethical goals of engagement (as described in the literature) and how this influenced (influences) the implementation of the activities, and the re-defining of the specific goals of the engagement activities.
- The consideration, facilitated by the ToC process, of the need for the data collection to include the voices of researchers, signifies (possibly?) a shift towards a more holistic understanding among practitioners, of the two-way nature of engagement. This in itself would appear to be a very valuable outcome of the process which could be emphasised.
- The shift from workshopping overall ethical programme goals, to defining specific goals of individual activities, highlights the possibly diverse range of potential goals/outcomes of engagement, some intended and some unintended. This could highlight the importance of including qualitative methods within the evaluation methods, so that the evaluation itself continues to contribute to the practitioners’ understanding of the range of possible outcomes.

 

 Overall, this article provides very important practical example of how ToC has been applied to evaluating community engagement. As such makes a valuable contribution to the engagement literature.

I have read this submission. I believe that I have an appropriate level of expertise to confirm that it is of an acceptable scientific standard.

# Peer review: Referee response for version 1

**DOI:** 10.21956/wellcomeopenres.14989.r30664

**Published:** 2018-02-15

**Authors:** Phaik Yeong Cheah

**Affiliations:** 1 Mahidol-Oxford University Tropical Medicine Research Unit, Faculty of Tropical Medicine, Mahidol University, Bangkok, Thailand; 2 University of Oxford, Oxford, UK

The paper describes the experiences of the MLW research group using theories of change to design monitoring and evaluation strategies.

 There is a paucity of literature on evaluation in general. I applaud the authors for writing up their experiences and struggles with this field of work as it would help others working in the same area.

 My specific comments are as follows:

Introduction

 Please define what you mean by “engagement” in the context of this paper.

 The term used in the paper is “community engagement in research”. Does this include community engagement in science?

Methods

 Please briefly describe the exhibition project for example, what type of exhibits are available, are they permanent, is it open all the time or visitors need to make an appointment, are the exhibits mainly biomedical or do they include physics, chemistry etc?

 Please briefly describe or include a reference to the “Community Advisory Group”

Results

 You talked about time being an issue. Suggest commenting on the proportionality (time, money, resources) of M&E activities compared to engagement activities themselves.

I have read this submission. I believe that I have an appropriate level of expertise to confirm that it is of an acceptable scientific standard.
